# Effect of Zeolite Supplementation on Gene Expression in the Intestinal Mucosa in the Context of Immunosafety Support in Poultry

**DOI:** 10.3390/genes13050732

**Published:** 2022-04-22

**Authors:** Aleksandra Dunislawska, Jakub Biesek, Mirosław Banaszak, Maria Siwek, Marek Adamski

**Affiliations:** 1 Department of Animal Biotechnology and Genetics, Faculty of Animal Breeding and Biology, Bydgoszcz University of Science and Technology, Mazowiecka 28, 85-084 Bydgoszcz, Poland; siwek@pbs.edu.pl; 2 Department of Animal Breeding and Nutrition, Faculty of Animal Breeding and Biology, Bydgoszcz University of Science and Technology, Mazowiecka 28, 85-084 Bydgoszcz, Poland; jakub.biesek@pbs.edu.pl (J.B.); miroslaw.banaszak@pbs.edu.pl (M.B.); adamski@pbs.edu.pl (M.A.)

**Keywords:** aluminosilicate, cecum, immune response, one health, poultry

## Abstract

Zeolite is an effective and non-toxic silicate mineral. Its properties are widely used in industry due to its sorption and ion exchange properties. Due to its excellent chemical properties, it has also great potential in poultry production as a food additive or supplement to bedding. This is of great importance for the biosafety and hygiene of production. The study aimed to analyse the effects of simultaneous application of zeolite to feed and bedding on production parameters and expression of genes related to intestinal tightness, organism defence, and immune response. Male Ross 308 broiler chickens were used in the experiment. In the experimental group, an external factor in the form of a powdery zeolite was used for feed and pelleted bedding. On the day of slaughter, the caecal mucosa was collected for gene expression analysis. We showed no significant changes in the tissue composition of the carcasses, but zeolite had a beneficial effect on the carcass yield. The analysis of the immune gene panel showed a significant increase in the expression of the interleukins and interferons genes. We have demonstrated the effect of zeolite on the improvement of the intestinal barrier and increasing the tightness of the intestines. There were no changes in gene expression related to the host’s defence against infections; therefore, based on the obtained results, it was concluded that zeolite can be considered an immunomodulating factor of the immune system.

## 1. Introduction

Zeolite is one of the silicate minerals that is considered to be effective and non-toxic. Its properties are widely used in the industry because it is characterized by a high sorption and ion exchange capacity [1]. Due to their excellent chemical properties, zeolites have great potential in poultry production. Zeolites might be used as a food additive and supplementing bedding. The introduction of zeolite on poultry farms affects productivity, and carcass quality, and also reduces environmental pollution [1]. Aluminosilicates could be used as an additive to the litter which resulted in the neutralization of ammonia [2]. The second option is to use zeolites for feed as an additive to improve nutrients consumption and growth [3]. It has been demonstrated an impact of zeolite on the carcass features and physiological status [4]. Our previous study has shown that the application of aluminosilicates to the litter and fodder has an impact on production parameters, especially: a lower water-holding capacity in the breasts. It has also a positive effect on weight gain, and feed conversion ratio. Such supplementation also influenced the expression profile of genes related to the immune system: Th2-type cytokines, pro-inflammatory and antiviral [5]. 

The quality of meat is a set of traits proving the usefulness of the raw material for further technological processing and consumption [6]. It is also closely related to production efficiency [7]. Meat quality is under the influence of production, including weight gain and feed consumption. Other important factors which have a significant impact on meat quality are basic production indicators, nutrition, and the environmental conditions of broiler rearing [8]. The awareness of production safety is constantly growing. The safety of poultry production is defined as biosecurity and the health status of birds [9,10]. Biosecurity is an element of good production practices that ensures an adequate level of hygiene in the broiler house [11]. Building hygiene includes good quality litter, clean air and odour gas indicators, and appropriate temperature and moisture content of the litter [12]. Inadequate hygienic conditions can lead to an unfavourable physiological condition of the birds’ organism and, over time, impair the immune system [13]. Ultimately, this may result in a deteriorated quality of the meat [14].

The study aimed to analyse the effect of simultaneous application of zeolite to feed and bedding on production parameters and expression of genes related to intestinal tightness, organism defence, and immune response. Research in this direction is of great importance for sustainable poultry production and the support of ecological strategies. The presented research is in line with the OneHealth concept. The discussed issue assumes an innovative approach to potential as well as existing risks that arise through animal-human-ecosystem interaction [2].

## 2. Materials and Methods

### 2.1. Animals and Rearing Period

Male Ross 308 broiler chickens were used in the experiment. The rearing lasted 42 days. One-day-old chicks were divided into two equal groups of 100 each. Every group was divided into 10 replicates composed of 10 birds. Group C was the control group where broiler chickens were reared according to the standard conditions. In group Z (experimental group), an experimental factor in the form of a powdery zeolite was used for feed and pelleted bedding. The chemical composition of the zeolite is shown in Table 1, based on the supplier’s declaration. The chickens were kept in 1 × 1 m pens, by the rearing standards (max. 39 kg per 1 m^2^ of the surface). The room was fully controlled with no natural light. The temperature in the building on the first day was 30 °C (28 °C—floor temperature, 32 °C—air temperature) and gradually decreased to 20 °C in the 4th week of rearing. The average humidity was 60–65%. The lighting on day 1 was 23 h. Along the time of rearing, 6 h of darkness were provided. In the last 3 days before slaughter, the lighting schedule was used as at the beginning. The feeding scheme of the chickens was divided into three periods (starter, grower, and finisher) and the feed mixtures were complete (commercial). The feed met the requirements for broiler chickens. The feed was isogenic and isocaloric. Zeolite was added at the stage of compound feed production in a feed mill. Zeolite in the experimental group was added to the feed at the level of 0.5%. In turn, the addition to the pellet bedding was provided during rearing, where 0.650 kg (in total) of the powdery mineral was used per 1 m^2^. Zeolite was added on the 1st (starting the experiment) at 0.250 kg/m^2^, and then 0.200 kg/m^2^ on days 14 and 22 (feed change). 

### 2.2. Growth Performance and Slaughter Yield

During the rearing, the production results were monitored. The broiler chickens were weighed on the 1st, 14th, 22nd, and 42nd days of rearing (at the times of the feed change). Each chick was weighed twice (BW). The feed intake (FI) of the individual pens was recorded daily (calculated for each feeding period). Based on the obtained data, the weight gain (BWG) for individual rearing periods and the feed conversion ratio (FCR) were calculated. After the rearing was completed (day 42), 10 birds were randomly selected from each group and slaughtered following the standards and principles of humane handling of the birds during slaughter. The slaughter was performed by qualified persons. The slaughter yield of the chickens was calculated based on the percentage ratio of the carcass weight to the pre-slaughter weight (Radwag, Radom, Poland). Then, a simplified dissection was performed, where the pectoral muscles (major and minor), leg muscles (thigh and drumstick, trimmed) were separated, as well as the skin with subcutaneous fat and abdominal fat. The percentage share in the carcasses was calculated. Based on the collected data on growth performance and slaughter yield, statistical calculations were made (Statistica, 13.3. Statsoft, Kraków, Poland). The mean values for each of the examined features and the standard error of the mean (SEM) were calculated. Statistically significant differences between the control and experimental groups were verified using the Student’s *t*-test, assuming that the *p*-value was less than 0.05. 

### 2.3. Sample Collection and RNA Isolation

The cecal mucosa (n = 5 per group) was collected for gene expression analysis after slaughter. The cecum from each individual was cut lengthways after collection and rinsed in PBS. The mucosal layer was scraped with a glass slide. The collected tissues were fixed in RNA stabilizing buffer (RNA fix; EURx, Gdansk, Poland). Each tissue was homogenized in 1 mL of TRIzol reagent (MRC, Cincinnati, OH, USA) by using a TissueRuptor homogenizer (Qiagen GmbH, Hilden, Germany). 200 μL of chloroform was added to the homogenate, shaken, and centrifuged (12,000 rpm, 15 min). The aqueous phase with the isolated RNA was collected. RNA was additionally purified using a commercial set- Universal RNA Purification Kit (EURx, Gdansk, Poland) according to the manufacturer’s instructions. RNA was eluted in a volume of 50 μL of nuclease-free water. Qualitative and quantitative control of RNA was performed using a 2% agarose gel electrophoresis and spectrophotometer (Nanodrop 2000; Thermo Scientific, Wilmington, NC, USA). The RNA was stored at −20 °C, as recommended by the manufacturer of the isolation set.

### 2.4. Gene Panel Selection 

The gene panel for expression analysis was selected based on previous experience [5,15,16] and gene function. To avoid the randomness of genes, co-expression analysis was performed. The extended functional analysis was performed for proteins encoded by genes using String software (multiple proteins for *Gallus gallus*) [3]. The correlation between the expression of particular genes conditioning the given protein was estimated on the basis of databases and experiments in the String database. A network was created to show the current interaction between the proteins, and functional enrichments in the network (GeneOntology (GO); biological process) were generated.

### 2.5. Relative Gene Expression in Cecal Mucosa

Isolated RNA was reverse transcribed to cDNA (ThermoScientific, Maxima First Strand cDNA Synthesis Kit for RTqPCR; Thermo Scientific, Vilnius, Lithuania) according to the manufacturer’s protocol. The qPCR reaction was performed using the following reaction mixture: Maxima SYBR Green qPCR Master Mix (Thermo Scientific, Vilnius, Lithuania), 140 ng of cDNA, 1 μM of forwarding primer and 1 μM of the reverse primer. Primer sequences were derived from the literature data and our previous published scientific reports [5]. The geometric mean of two independent reference genes (*ACTB* and *G6PDH*) was used. The primer sequences are presented in Table 2. LightCycler 480 instrument II (Roche Diagnostics, Basel, Switzerland) was used to carry out the thermal program of reaction. The program consisted of initial denaturation (95 °C, 20 min) followed by 40 cycles of amplification (15 s, 95 °C), annealing (20 s, 58 °C—melting temperature for each pair of primers; expect for IL12—65 °C) and elongation (20 s, 72 °C). Each qPCR reaction was performed in duplicate technical repetitions.

## 3. Results

### 3.1. Growth Performance and Slaughter Yield

The addition of zeolite (Table 3) showed a statistically significantly higher body weight (BW) of chickens on the 22nd day of rearing and the 42nd day, (*p* = 0.019; *p* < 0.001, respectively). During the feeding period of chickens for fattening with grower and finisher feed, statistically, significantly higher body weight gain (BWG) was demonstrated in group Z compared to group C (*p* = 0.007; *p* < 0.001; *p* < 0.001, respectively). Despite significant differences in BW and BWG between groups, no significantly higher feed intake (FI) was found throughout the rearing period. At the finisher period, a significantly lower feed conversion ratio (FCR) was shown in the chickens from group Z compared to group C (*p* = 0.010).

Table 4 presents the results concerning the slaughter yield of broiler chickens. There were no statistically significant differences between the groups in the examined features (*p* > 0.05). Quantitatively higher slaughter yield was found in group Z, by 0.71% than in group C. The difference was associated with a higher share of leg muscles (by 1.86% in Z than in group C) and fatness (0.64% more of skin with subcutaneous fat and 0.31% more of abdominal fat). The share of breast muscles was lower by 1.9% in group Z than in group C.

### 3.2. Gene Panel Selection

**Figure 1:**
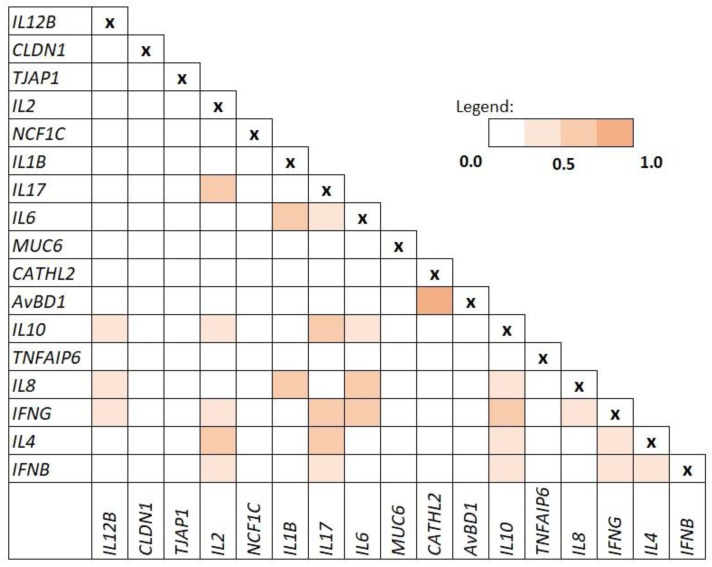
Co-expression matrix (level of confidence that presented proteins encoded by analysed genes are functionally associated; based on String software).

The selection of the gene panel was made based on gene functions and interactions. As evidenced by in silico analysis, strong co-expression exists between the genes encoding the AvBD1 and CATHL2 proteins. The most abundant co-expression with other genes is shown by IL2, IL17, and IL10. The co-expression matrix is shown in Figure 1. 

**Figure 2:**
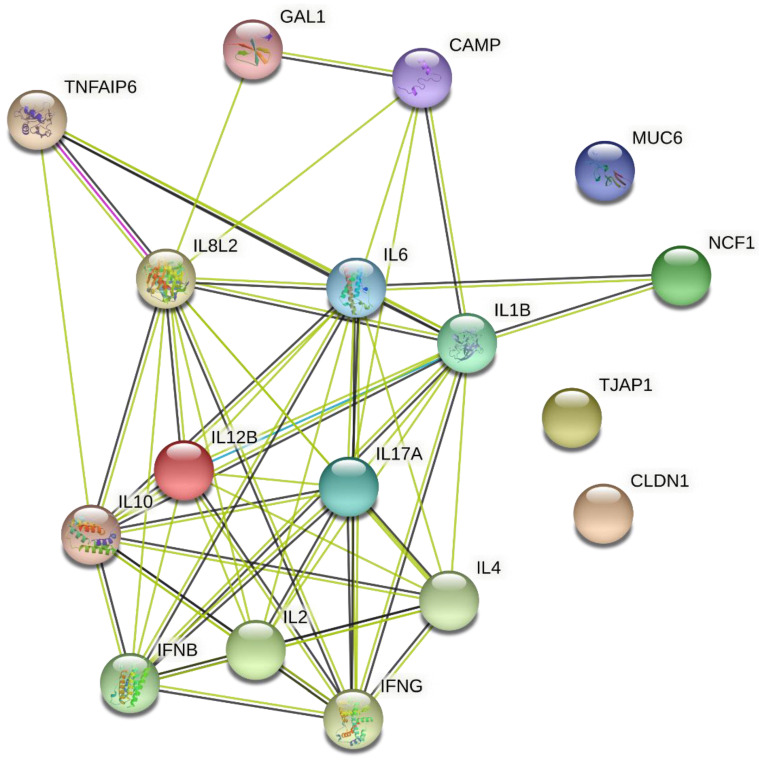
Analysis of the relationship (network) between proteins encoded by genes whose expression was analysed in the current study. Lines represent protein-protein associations. Lines of interactions according to String software: light blue—from curated databases; pink—experimentally determined; dark green—gene neighbourhood; red—gene fusions; dark blue–gene co-occurrence; light green–text mining; black—co-expression; violet—protein homology.

The intensity of the colour indicates the level of confidence that proteins are functionally associated. For evaluation, the overall expression data of *Gallus gallus* is taken into account. Figure 2 shows an analize of the relationship between proteins encoded by genes whose expression was analysed in this study.

Associations between proteins can be specific and meaningful. This interaction shows that proteins collectively contribute to a common function; however, this does not necessarily mean that they are physically related. 

The analysis of the functions of the selected genes allowed for the definition of GO terms. Selected genes perform functions such as defence response (11 genes), immune system process (11 genes), response to other organisms (10 genes), and also immune response (9 genes). The terms of GO defined based on biological processes in which selected genes are involved are presented in Table 5. 

### 3.3. Gene Expression in Cecal Mucosa

**Figure 3:**
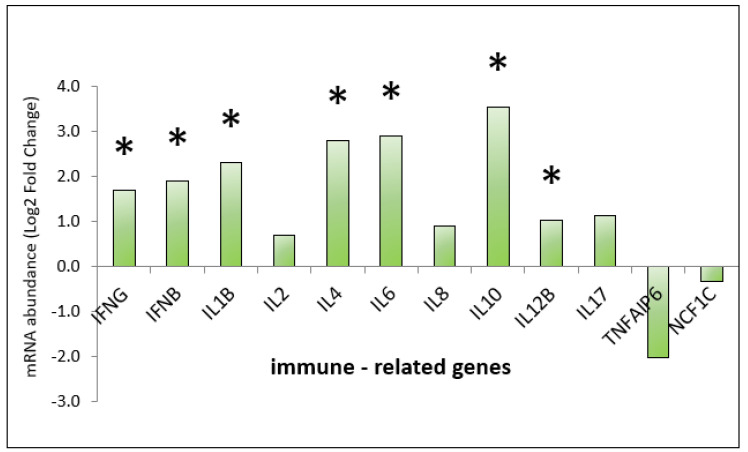
Relative gene expression of immune-related genes in cecal mucosa in chickens supplemented with zeolite in feed and bedding. Statistical analysis consisted of comparing the experimental groups with the control group using Student’s *t*-test (* for *p*-value < 0.05).

**Figure 4:**
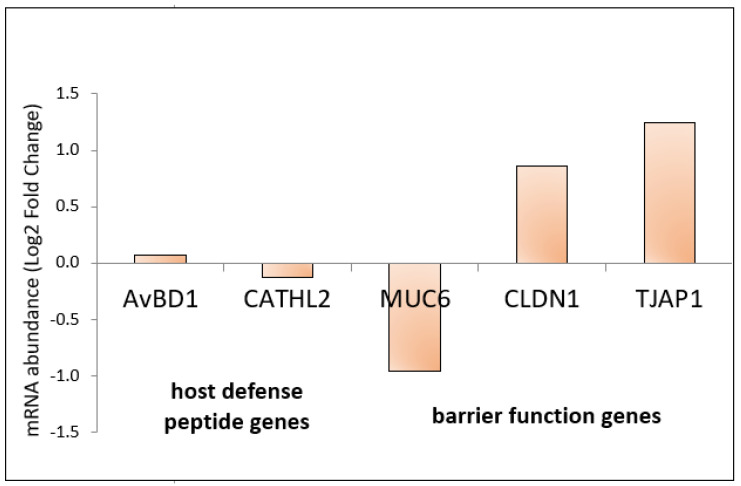
Relative gene expression of host defence peptide genes and barrier function genes in cecal mucosa in chickens supplemented with zeolite in feed and bedding. Statistical analysis consisted of comparing the experimental groups with the control group using Student’s *t*-test.

The relative gene expression in the cecal mucosa showed statistically significant upregulation (*p* < 0.05) in 9 out of 12 analysed immune-related genes: *IFNG*, *IFNB*, *IL1B*, *IL2*, *IL4*, *IL6*, *IL8*, *IL10*, *IL12B*. These results are presented in Figure 3. The level of expression of genes related to the intestinal barrier increased numerically, but these results are not statistically significant (Figure 4). No statistical and numerical changes in the expression of host defence genes were observed (Figure 4).

## 4. Discussion

The study was undertaken to address the possibilities to improve broiler chicken health status. The presented solution is the utilization of zeolite in the feeding strategy and chicken maintenance. We have verified the impact of zeolite supplementation on the immune status of poultry-based on the expression of genes related to the immune response and intestinal barrier and improvement of production parameters. 

Our research showed a significant effect of zeolite supplementation on chickens’ growth. Despite elevated body weight gains, no increased feed consumption was found. Liu et al. [23] found that the use of hydrated sodium calcium aluminosilicate at the level of 3.0 g/kg affected the increase in daily weight gain and feed consumption ratio. Similarly, the use of 1–2% zeolite in fodder for turkeys (both sexes) affected the production indicators [24]. The addition of minerals in the feed may affect the better utilization of nutrients, which in turn stimulates their better growth [25]. Opposite effects were presented by Shariatmadari. These authors showed the absence or the adverse effect of the use of aluminosilicates [26]. These discrepancies might be explained by the number of substances supplied into fodder and other factors that cause interactions. Usage of natural minerals in poultry production should be done concerning the species and direction of the use of the animals, and even the composition of the feed. The aluminosilicates may show various metabolic and molecular effects when combined with other additives [27]. Semenenko et al. [28] showed that a 2% addition of bentonite improved metabolic homeostasis in broiler chickens. Improvement in the production of broiler chickens was found in the experiments performed by Zhou et al. [1]. The 2% addition of zeolite in combination with attapulgite (1:1) increased the secretion of digestive enzymes, improved the digestibility of nutrients, and had a beneficial effect on the health of the intestines.

We showed no significant changes in the tissue composition of the carcasses, including the slaughter yield, the share of breast and leg muscles, as well as skin with subcutaneous fat and abdominal fat. Similarly, the use of 1% zeolite did not show any significant differences in the slaughter yield of chickens [4]; however, a higher zeolite supplementation (2%) had a beneficial effect on the carcass yield. This might suggest that the small dose (i.e., 0.5%) of aluminosilicates in the feed will not affect the characteristics of the carcass. 

Gene expression analysis was performed in three gene panels related to immune response, host defence, and intestinal barrier functions. These analyses were performed on the mucosa of the caecum. In birds, the caecum is double and symmetrical. They are located at the junction of the ileum and large intestines. The intestines of birds contain gut-associated lymphoid tissue (GALT), which consists of lymphoid cells dispersed throughout the intestinal epithelial tissue and organized lymphatic structures associated with the intestinal lamina propria, Peyer’s patches, and caecal tonsils. Within these structures, various types of immune cells activate diverse immune responses [29]. The caecum of poultry species is also the location of a complex microbes community; it plays a vital role in preventing pathogens from colonizing the gut, processing nutrients, and, most importantly, detoxifying the gut [30]. The cecum, due to the extensive population of microorganisms and the longer transit time of the chyme, is the main region of bacterial fermentation, as well as the site of pathogens colonization [31].

The analysis of the immune gene panel showed a significant increase in the expression of the interleukins and interferons genes. Our previous study proved that aluminosilicates are a gentle stimulant of the immune system [5]. A moderate degree of immune system stimulation is essential for animal production. Productivity may be adversely affected in the event of an excessive immune response [32]. The analysis of production parameters shows that the indicated change in the expression of genes related to the immune system does not harm growth; thus, zeolite can be considered a good immunomodulator to enhance the immune response in poultry maintaining broilers’ performance at the same level. This is an important condition of animal production immunosecurity [33]. Interferons are involved in the innate immune response against viruses in birds [34]. The level of *IFNG* and *IFNB* expression detected in our study might indicate sufficient protection for the host against viral pathogens. Cytokines are protein signalling compounds. They are the main mediator of the immune response and also control many cellular functions. The pro-inflammatory cytokine *IL1B* in the intestines is secreted into the intestinal lumen and is an important mediator of intestinal inflammation. Changes in its expression in intestinal mucosa may be influenced by commensal intestinal microbiota [35]. Therefore, it might be implied that the expression of *IL1B* shown in this study is related to microbial activity in the intestines. The immunostimulatory properties of the zeolite may also be demonstrated by the increased expression of the pro-inflammatory cytokine *IL12* and the anti-inflammatory cytokine *IL10*. These interleukins play an important immunoregulatory role in the host’s defence. They are produced primarily by antigen-presenting cells activated by the pathogen [36]. As demonstrated by Susta et al. [37] *IL2* in chickens activates T cells and may affect the replication and pathogenesis of the Newcastle disease virus. Increased expression of this gene during viral replication significantly lowered the viral load in blood, spleen, and secretions. Literature reports show that *IL4* significantly influences the regulation of macrophage functions in chickens [38]. *IL6* is a key cytokine in intestinal inflammation, both as a pro-inflammatory factor and as a regeneration stimulant [39].

The results of genes related to the intestinal barrier are consistent with our previous report, where aluminosilicates (zeolite and halloysite) were supplied in feed and litter [5]. A numerically significant increase of *CLDN1* and *TJAP1* expression in the intestinal mucosa may indicate the beneficial effect of zeolite on sealing the intestinal barrier. The proteins encoded by these genes act as a major component of tight junction [40]. These connections ensure the tightness of the barrier between the intestinal microbiota and the host organism, protecting the organism against endotoxemia. A properly functioning and shaped intestinal epithelium acts as a selectively permeable barrier that permeates nutrients, water, and electrolytes; it also effectively protects against the entry of toxins, antigens, and pathogenic bacteria [41], and is also a place of communication between microorganisms and the host’s immune system. Literature data show that the unsealing of the intestinal epithelium barrier is a factor determining the inflammation of the gastrointestinal tract [42].

The analysis of the expression of the *AvBD1* (defensin) and *CATHL2* (cathelicidin) genes showed no significant changes. Defensins are the main family of defence peptides in the host organism, which are expressed primarily in epithelial cells. They exhibit broad antimicrobial activity as well as multilateral immunostimulating and immunomodulating functions. Their main function is to protect the host against bacterial, viral, and fungal infections. They also have the ability to kill bacteria and inhibit their growth. Some literature reports mention the unfavourable function of defensins in specific biological conditions, by promoting bacterial infections [43]. In turn, cathelicidins, host protective proteins, play an important role in innate and acquired immunity. Like defensins, they can eliminate pathogens and modulate the immune response [44]. The lack of changes in gene expression of *AvBD1* and *CATHL2* in the current study may indicate a favourable health status of individuals, so it was not necessary to defend the organism against pathogens.

## 5. Conclusions

We can conclude that the use of a lower dose of zeolite did not show any significant differences in the slaughter yield of chickens, but a higher zeolite supplementation had a beneficial effect on the carcass yield. The analysis of the immune-related gene panel showed a significant increase in the expression of the interleukins and interferons genes. It can be concluded that zeolite can be considered an immunomodulating factor that enhances the immune response. In this way, the organism is protected without excessive stimulation of the immune system, which could translate into negative production parameters. This is an important step toward research into the immunosafety of poultry. It has been also shown the effect of zeolite on tight junctions and increasing intestinal tightness, which has a beneficial effect on maintaining the appropriate immune status of the organism. 

## Figures and Tables

**Table 1:** Chemical composition of zeolite.

Components (%)	Zeolite
SiO_2_ (silicon dioxide)	71.30
Al_2_O_3_ (aluminium oxide)	13.10
CaO (calcium oxide)	5.20
K_2_O (potassium oxide)	3.40
Fe_2_O_3_ (iron (III) oxide)	1.90
MgO (magnesium oxide)	1.20
Na_2_O (sodium oxide)	1.30
TiO_2_ (titanium oxide)	0.30
Si/Al (silicon/aluminium)	5.40
Clinoptilolite	84.00
Cristobalit	8.00
Mica clay	4.00
Plagioclases	3.50
Rutile	0.20
Data based on the supplier declaration	

**Table 2:** Primer sequences used in RT-qPCR reaction (F—Forward primer; R—Reverse primer).

Gene	Name	Primer Sequences	Ref.
*ACTB*	Actin β	F: CACAGATCATGTTTGAGACCTT R: CATCACAATACCAGTGGTACG	Adapted from Ref. [17]
*G6PDH*	Glucose-6-phosphate dehydrogenase	F: CGGGAACCAAATGCACTTCGT R: GGCTGCCGTAGAGGTATGGGA	Adapted from Ref. [17]
*IFNG*	Interferon γ	F: ACACTGACAAGTCAAAGCCGC R: AGTCGTTCATCGGGAGCTTG	Adapted from Ref. [18]
*IFNB*	Interferon β	F: ACCAGATCCAGCATTACATCCA R: CGCGTGCCTTGGTTTACG	Adapted from Ref. [19]
*IL1B*	Interleukin 1 β	F: GGAGGTTTTTGAGCCCGTC R: TCGAAGATGTCGAAGGACTG	Adapted from Ref. [15]
*IL2*	Interleukin 2	F: GCTTATGGAGCATCTCTATCATCA R: GGTGCACTCCTGGGTCTC	Adapted from Ref. [20]
*IL4*	Interleukin 4	F: GCTCTCAGTGCCGCTGATG R: GGAAACCTCTCCCTGGATGTC	Adapted from Ref. [19]
*IL6*	Interleukin 6	F: AGGACGAGATGTGCAAGAAGTTC R: TTGGGCAGGTTGAGGTTGTT	Adapted from Ref. [21]
*IL8 (IL8L2)*	Interleukin 8	F: AAGGATGGAAGAGAGGTGTGCTT R: GCTGAGCCTTGGCCATAAGT	Adapted from Ref. [19]
*IL10*	Interleukin 10	F: CATGCTGCTGGGCCTGAA R: CGTCTCCTTGATCTGCTTGATG	Adapted from Ref. [22]
*IL12 (IL12B)*	Interleukin 12	F: TTGCCGAAGAGCACCAGCCG R: CGGTGTGCTCCAGGTCTTGGG	Adapted from Ref. [18]
*IL17*	Interleukin 17	F: CCGTCTTCTGCTGAGAGGAGTG R: ACCGTTGTTCCGTCCCATCAC	Adapted from Ref. [20]
*TNFAIP6*	Tumor necrosis factor-inducible gene 6 protein	F: CTGGCTGTCCCTGTGTGATT R: TCAGGTGCTATTGCTGCGAG	Adapted from Ref. [5]
*NCF1C*	Neutrophil Cytosolic Factor 1C	F: CTGTGGATGGTGTCACCGAA R: TGCCATTCTCACAGCCCTAC	Adapted from Ref. [5]
*AvBD1 (GAL2)*	Avian β-defensin 1	F: AAACCATTGTCAGCCCTGTG R: TTCCTAGAGCCTGGGAGGAT	Adapted from Ref. [15]
*CATHL2 (CAMP)*	Cathelicidin	F: AGGAGAATGGGGTCATCAGG R: GGATCTTTCTCAGGAAGCGG	Adapted from Ref. [15]
*MUC6*	Mucin 6	F: TTCAACATTCAGTTCCGCCG R: TTGATGACACCGACACTCCT	Adapted from Ref. [15]
*CLDN1*	Claudin 1	F: TCTTCATCATTGCAGGTCTGTC R: AACGGGTGTGAAAGGGTCAT	Adapted from Ref. [15]
*TJAP1*	Tight junction-associated protein 1	F: AGGAAGCGATGAATCCCTGTT R: TCACTCAGATGCCAGATCCAA	Adapted from Ref. [15]

**Table 3:** Growth performance of broiler chickens.

Item ^1^	Group ^2^		SEM ^3^	*p*-Value
	C	Z		
BW (g)				
1-day old chicks	46.69	46.64	0.28	0.933
14 day	499.65	499.67	3.25	0.998
22 day	1598.25 ^b^	1656.05 ^a^	12.73	0.019
42 day	2822.65 ^b^	3088.16 ^a^	38.92	<0.001
BWG (g)				
1–13 days	452.96	448.16	3.51	0.509
14–21 days	1098.60 ^b^	1143.92 ^a^	8.94	0.007
22–42 days	1224.40 ^b^	1432.34 ^a^	33.47	<0.001
1–42 days	2775.96 ^b^	3024.42 ^a^	37.25	<0.001
FI (g; per bird)				
1–13 days	530.00	490.00	10.98	0.067
14–21 days	1900.00	1965.00	58.16	0.590
22–42 days	1615.00	1585.00	75.50	0.266
1–42 days	5126.54	5556.40	202.06	0.300
FCR (kg/kg)				
1–13 days	1.17	1.09	0.02	0.075
14–21 days	1.73	1.72	0.06	0.888
22–42 days	1.32 ^a^	1.11 ^b^	0.06	0.010
1–42 days	1.85	1.84	0.07	0.932

^a,b^ significance between control and zeolite groups; *t*-test, *p*-value < 0.05; ^1^ BW, body weight; BWG, body weight gain; FI, feed intake; FCR, feed conversion ratio; ^2^ C, control group; Z, zeolite group; ^3^ SEM, standard error of the mean.

**Table 4:** Slaughter yield of broiler chicken.

Item (%)	Group ^1^		SEM ^2^	*p*-Value
	C	Z		
Slaughter yield	78.03	78.74	0.34	0.300
Breast muscles	32.73	30.83	0.52	0.068
Leg muscles	19.75	21.61	0.48	0.053
Skin with subcutaneous fat	8.29	8.93	0.26	0.231
Abdominal fat	0.96	1.27	0.10	0.124

no significance between control and zeolite groups was found; *t*-test, *p*-value > 0.05; ^1^ C, control group; Z, zeolite group; ^2^ SEM, standard error of the mean.

**Table 5:** Functional enrichment in gene network based on Gene Ontology terms (Biological Process) created by String software.

Term ID	Term Description	Observed Gene Count	Background Gene Count	Strength	False Discovery Rate
GO:0006952	Defence response	11	588	1.23	2.44 × 10^−8^
GO:0051707	Response to another organism	10	583	1.19	4.42 × 10^−7^
GO:0002376	Immune system process	11	1140	0.94	4.56 × 10^−6^
GO:0006955	Immune response	9	605	1.13	5.51 × 10^−6^
GO:0098542	Defence response to another organism	8	423	1.24	7.62 × 10^−6^
GO:0009617	Response to bacterium	6	266	1.31	2.70 × 10^−4^
GO:0001775	Cell activation	6	322	1.23	0.00068
GO:0006959	Humoral immune response	4	95	1.58	0.0025
GO:0034097	Response to cytokine	6	420	1.12	0.0027
GO:0045321	Leukocyte activation	5	271	1.23	0.0056
GO:0045918	Negative regulation of cytolysis	2	4	2.66	0.01
GO:0050727	Regulation of inflammatory response	4	150	1.39	0.0105
GO:0070673	Response to interleukin-18	2	5	2.56	0.0117
GO:0071345	Cellular response to cytokine stimulus	5	378	1.08	0.0204
GO:0080134	Regulation of response to stress	6	667	0.91	0.024
GO:0002274	Myeloid leukocyte activation	3	68	1.6	0.0255
GO:2000377	Regulation of reactive oxygen species metabolic process	3	72	1.58	0.0288
GO:0006954	Inflammatory response	4	219	1.22	0.0335
GO:0070887	Cellular response to chemical stimulus	8	1504	0.69	0.0335
GO:0050832	Defence response to fungus	2	12	2.18	0.037
GO:0006953	Acute-phase response	2	13	2.15	0.0411
GO:0042221	Response to chemical	9	2126	0.59	0.0499
GO:0048584	Positive regulation of response to stimulus	7	1186	0.73	0.0499
GO:0061844	Antimicrobial humoral immune response mediated by antimicrobial peptide	2	15	2.09	0.0499

## Data Availability

All the data, methods, and results of the statistical analyses are reported in this paper. We remain at your disposal in case of any questions.
